# Treatment of lymphocele with negative pressure wound therapy post inguinal mass excision: A case-report

**DOI:** 10.1016/j.ijscr.2019.11.017

**Published:** 2019-11-19

**Authors:** Caio Cesar Martins Focássio, Ricardo Augusto Bravo Gamboa, Luis Felipe Staut de Marco, Daniela Mina Fukasawa, Talita da Silva Parente, Vitor Leoni Boher Lopes Dornas

**Affiliations:** Department of Angiology/Vascular Surgery, Beneficência Portuguesa, Rua Maestro Cardim, 769, Bela Vista, Sao Paulo, SP, 01323-001, Brazil

**Keywords:** Lymphocele, Lymphatic fluid, Ligation of lymphatic vessels, Inguinal lymphocele, Lymphocele excision

## Abstract

**Introduction:**

A lymphocele is defined as an atypical collection of lymphatic fluid not bordered by distinct epithelial lining, which develops in anatomic compartments. Inguinal lymphocele is a common complication of surgery in the inguinal region, with an incidence ranging from 1 to 87 %. This report summarizes the management of an inguinal lymphocele post excision of an inguinal mass.

**Presentation of case:**

Herein, we present a case in which an inguinal lymphocele developed four months post excision of an inguinal mass, which was later diagnosed as lymphoma and treated with iliac chain radiation therapy. The conservative treatment with lymphocele drainage, compression dressing and prophylactic antibiotic was initially implemented. As the patient did not respond to conservative treatment, the surgical strategy consisted of excision of lymphocele associated with lymphatic ducts ligation. Negative pressure wound therapy completed the treatment.

**Discussion:**

Non-surgical treatment of lymphocele developing from lymphatic injure during groin dissection is not rarely unsuccessful. Surgical options include lymphocele excision with either ligation of the lymphatic ducts or lymphatic-venous shunts between afferent lymphatics and the collateral branch of great saphenous vein. Vacuum-assisted closure therapy assists the wound healing process by increasing blood flow, removing inhibiting factors of wound healing and decreasing the bacterial count.

**Conclusion:**

Inguinal lymphocele that is not reabsorbed or does not resolve with conservative treatment should be surgically treated. Lymphocele excision with ligation of lymphatic vessels, followed by negative pressure wound therapy appears to be a safe and effective approach.

## Highlights

• Safe and effective approach for treating inguinal lymphocele.
• Excision with ligation of lymphatic vessels.
• Negative pressure wound therapy.

## 1 Introduction

Lymphocele is defined as an atypical collection of protein-rich lymphatic fluid not bordered by distinct epithelial lining, which develops in anatomic compartments, with no inflammatory or granulomatous reaction at the leakage site [[1], [2], [3], [4], [5]].

Lymphoceles generally result from interruption to the lymphatic system that lead to nonstop drainage of the afferent lymphatics after surgery. Its formation has been described as a complication of surgeries performed in and near the femoral and inguinal vessels [1], amongst others. Inguinal lymphocele is a common complication of surgery in the inguinal region, with an incidence ranging from 1 to 87 % [3,[6], [7], [8]].

On clinical examination, overlying edema and erythema, and underlying mass can be observed [9]. Radiographic examination, needle aspiration with cytologic [1] and biochemical [10] analysis may be necessary for confirmation of lymphocele diagnosis. Small, uninfected collections are frequently asymptomatic, and an expectant approach to management is best adopted because lymph fluid collection will collateralize through alternative pathways, and lymphocele will spontaneously resorb [4].

Treatment becomes necessary by virtue of (i) infection, where antibiotics alone are often sufficient [11]; and (ii) size and compression, which require more aggressive approaches such as needle aspiration, percutaneous catheter drainage, and external or internal surgical drainage [9].

The aim of this paper was to report a case of inguinal lymphocele post inguinal mass excision and review the most important diagnosis features and treatment options for this condition. This manuscript has been reported in line with the SCARE criteria [12].

## 2 Presentation of case

A 72-year-old man presented to the hospital complaining about a large mass at the right inguinal region. Four months before, patient had a history of enlarged right inguinal volume. The inguinal mass was excised and later diagnosed as lymphoma, which was treated with 18 sessions of para-iliac ganglia radiotherapy. Three months later, patient underwent drainage of 300 mL lymph fluid.

On 27th November 2018, the patient was hospitalized for further examination and treatment of his symptoms. He denied systemic symptoms but referred anti-aesthetic complains and local discomfort. Physical examination revealed a soft large mass in the right side of the inguinal region, compatible with a lymphocele. The patient was referred to the vascular surgery team, which recommended oral prophylactic antibiotic therapy (Clavulin®, 1000 mg per day for 8 days, GlaxoSmithKline Inc., Ontario, Canada), drainage of lymph fluid, and associated therapy with compression dressing and micronized purified flavonoid fraction (Daflon, 2000 mg per day until patient discharge from hospital, Servier, France).

**Fig. 1:**
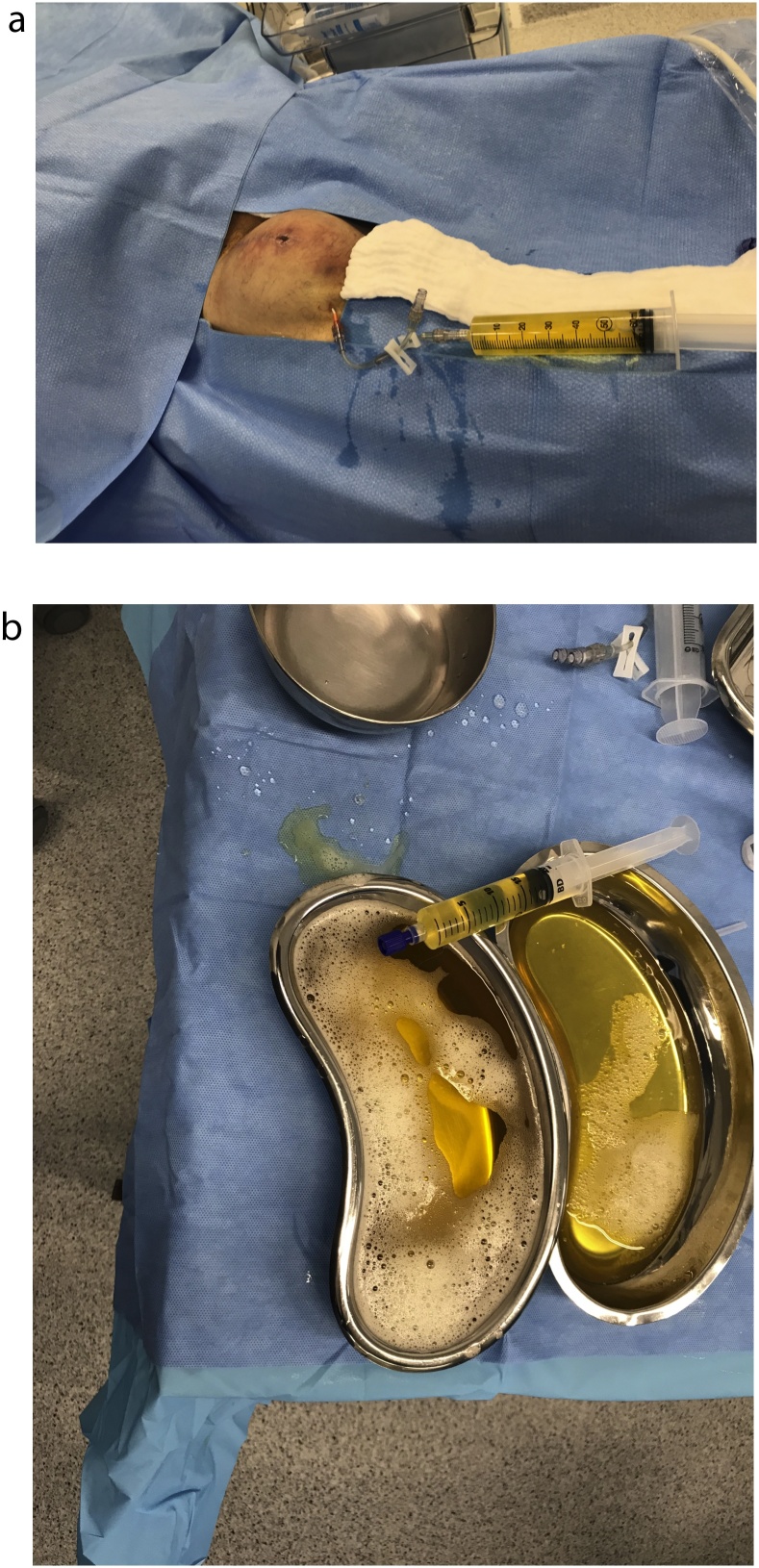
(a) Lymphocele drainage with 14G catheter. (b) Drained lymph fluid (728 mL).

On the next day, patient underwent catheter drainage of 728 mL lymph fluid (JELCO-W I.V. Catheter 14 G, Smiths Medical, Minneapolis, Minnesota, USA) (Fig. 1), followed by drainage catheter placement (Penrose drain 1”, Kent Elastomer Inc., Kent, Ohio, USA) with a Karaya pouch. Compressive bandaging was used, and patients started wearing two overlaid 30−40 mmHg compression stockings. Drained lymph fluid tested negative for bacterial infection.

Twenty-four hours later, patient was feeling well, stable, without complaints. Eighty mL of lymph fluid was collected in the Karaya pouch. Compression bandaging was changed for a clean one.

**Fig. 2:**
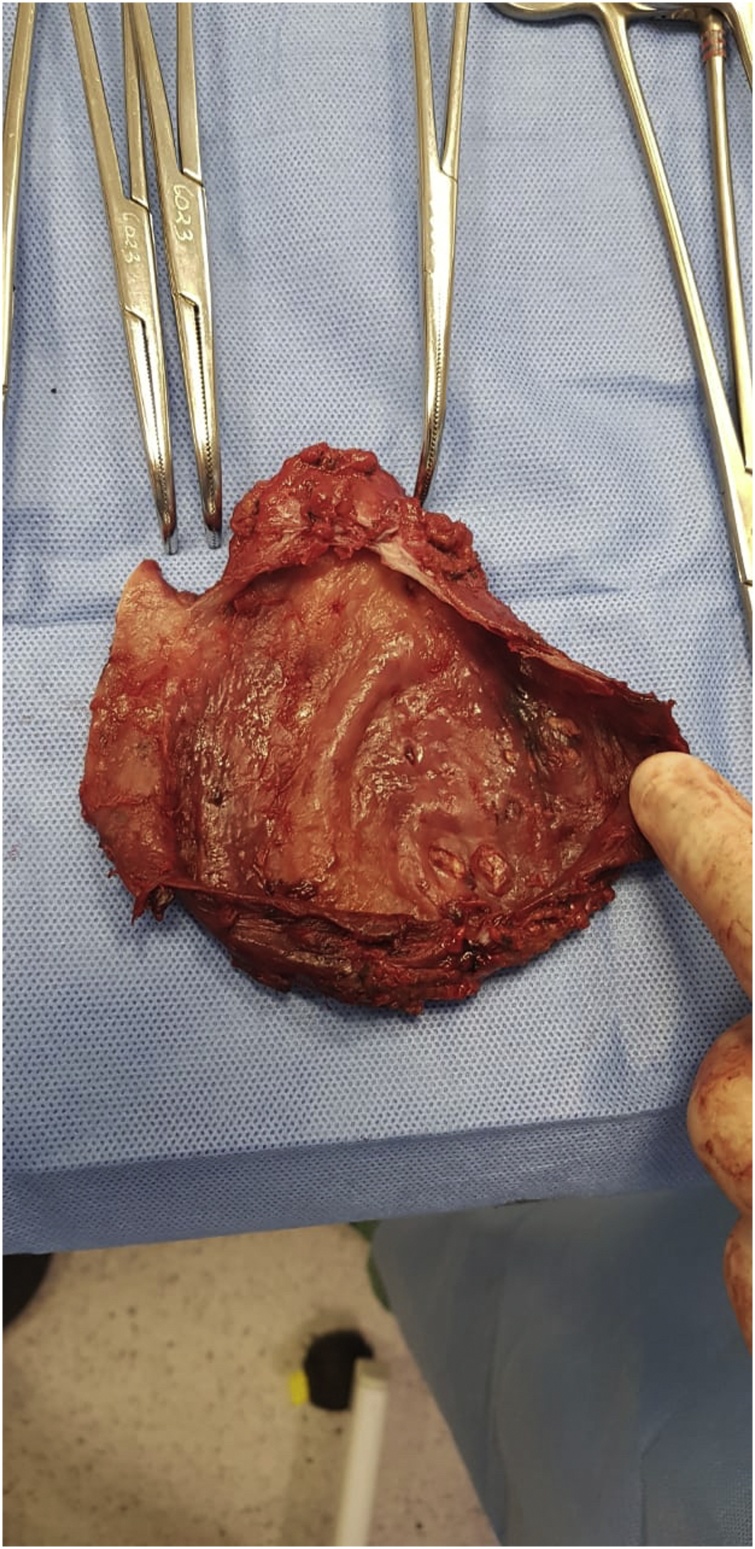
Excised lymphocele with its capsule.

**Fig. 3:**
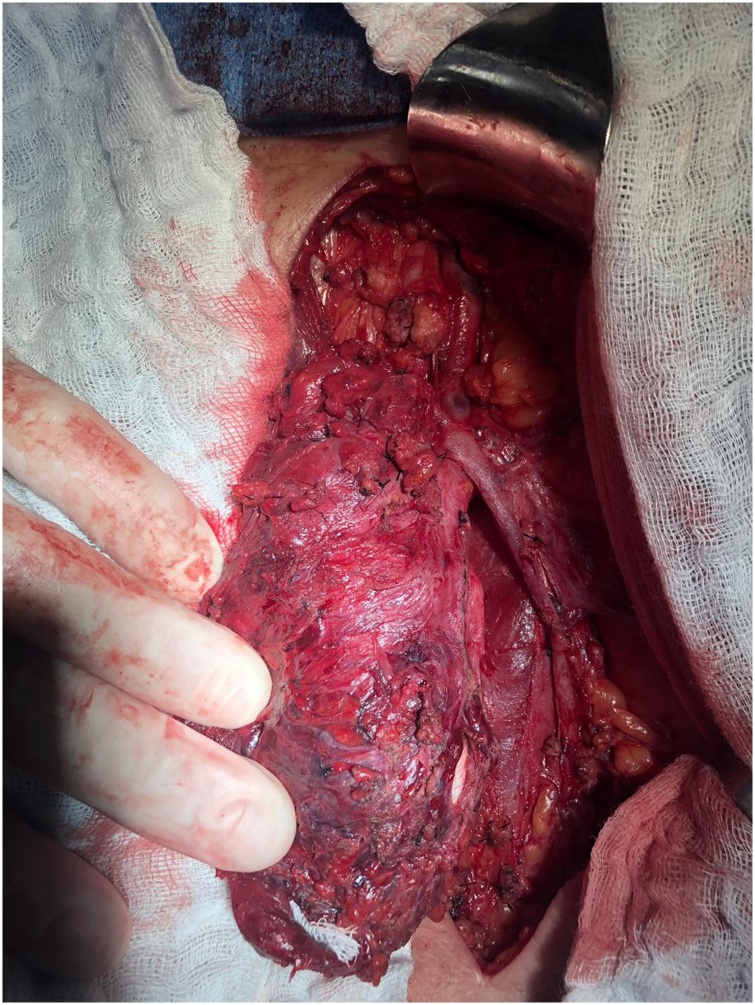
Ligation of lymphatics.

As the patient did not respond to conservative treatment, including multiple aspirations of lymph cavity and compression bandaging, surgical approach was adopted on December 5th. Surgical approach included complete excision of lymphocele with its capsule (Fig. 2) and lymphatic vessels ligation (Fig. 3). The surgery was completed in five hours, without intercurrences. Compressive bandaging and elastic bandage wrapping were later applied. Compression shorts and stockings were used to optimize lymphatic drainage and prevent lymphedema. Patient was admitted to intensive care unit for one day and followed up clinically and by ultrasonography as concerns lymphocele. Lymphocele capsule tested positive for bacterial infection (Staphylococcus caprae). Antibiotic therapy was changed according to the result of lymphocele capsule culture, which demonstrated bacterial sensitivity to Linezolid (Zivox, 600 mg per day for 6 days, Pfizer, NY, NY, USA,).

Five days later, bandaging was changed and a moderate serohematic secretion was noted. During the following days, secretion was drastically reduced and the patient was feeling well and asymptomatic.

**Fig. 4:**
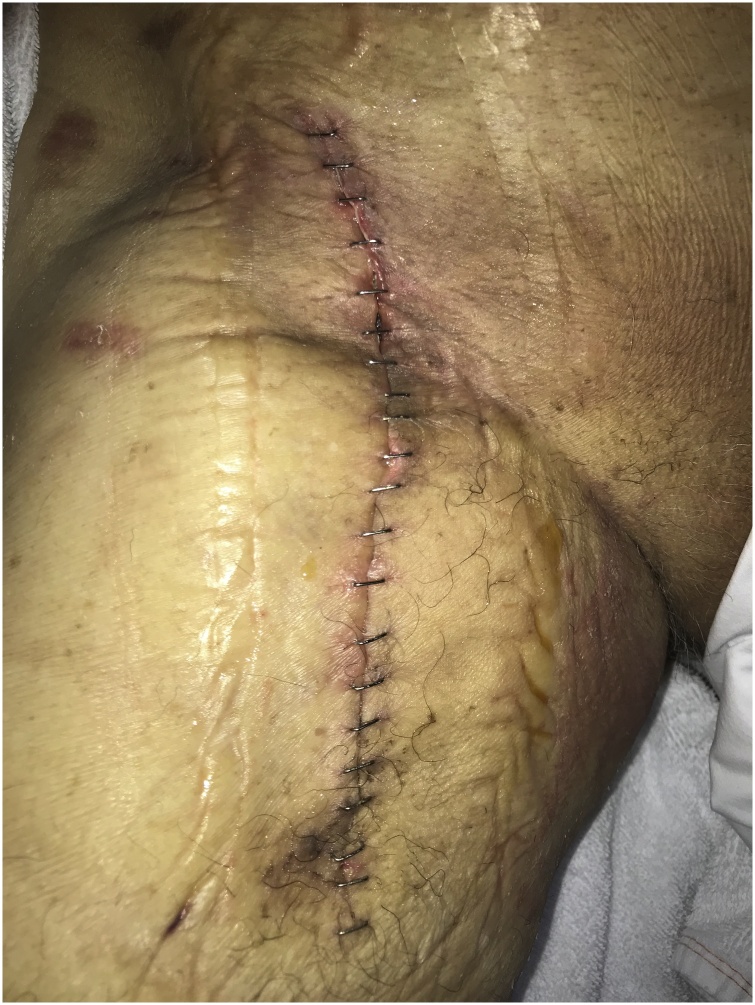
Closed skin incision after stapling technique.

On December 22nd, patient was submitted to another surgery by virtue of a postoperative wound disruption. Upon re-exploration, small lymphatic lakes were noted and drained. We also noted lymphorrhea in a small volume on right inguinal region, however, no obvious leakage point was found. Excess fibrin was noted and resected. Drainage perforations were occluded with 10 mL fibrin sealant. Povidone iodine in alcohol-based solution was used to wash operative wound and cause inflammatory reaction. Subcutaneous tissue approximation was performed and skin incision was closed with surgical stapling technique (Fig. 4), to avoid maceration. Three days post-surgery, negative pressure wound therapy (NPWT, Prevena™ Incision Management System, KCI), with intermittent pressure at −100 mmHg) was implemented.

On 29th December, NPWT system was changed for a fresh one. A small volume of lymph fluid was drained into the system. The patient was feeling well and asymptomatic, and was discharged from hospital with a scheduled return in two weeks.

On 16th January, the patient returned to the hospital for a scheduled NPWT system evaluation. There was no fluid collected in the system, and a small amount of fluid in the sponge. Wound presented coaptation of edges and optimal evolution. By inguinal ultrasound, we confirmed absence of liquid collection, including around the artery and femoral vein. Wound stiches were alternately removed. The patient was oriented to apply a simple dressing and topic antibiotic in the surgical wound (Rifocina® – Hoechst).

The patient returned to the hospital one week later for a scheduled evaluation. The patient complained no more about the mass sensation and discomfort referred before the treatment. The wound healed completely and the patient was discharged from hospital with no follow-up appointments booked.

## 3 Discussion

Lymphocele is usually a surgical complication that happens by virtue of damaged lymph flow channels, and may develop days to years after the injury [1]. The risk of lymphocele development correlates to the extent of lymphatic tissue removal during surgery [13]. Moreover, preoperative radiation therapy, heparin prophylaxis, and involvement of the lymph nodes by tumour [4] also predispose of lymphocele development. The importance of perioperative lymphatic stasis has been emphasized [5].

Herein, we reported a case of inguinal lymphocele post excision of an inguinal mass that was later diagnosed as lymphoma, which in turn was treated with iliac chain irradiation. It can be suggested that the development of this inguinal lymphocele occurred because of the inguinal mass excision that was not followed by ligation of the lymphatics, which caused the lymphatic ducts to remain opened, thus disrupting lymph flow. Additionally, irradiation contributed to worsening the scenario, as it interrupted iliac chain drainage just above the inguinal region that was already damaged.

The treatment was started conservatively with lymphocele aspiration, compression dressing and prophylactic antibiotic. However, the patient did not respond to conservative treatment, and a surgical intervention was needed, which consisted of lymphocele excision and lymphatic ducts ligation. Seventeen days post-surgery, the patient presented with a postoperative wound disruption, which was re-explored. Upon re-exploration, small lymphatic lakes were drained, and drainage perforations were occluded with fibrin sealant. The treatment was completed with the implementation of NPWT.

Inguinal lymphocele is a well-known complication after inguinal lymphadenectomy [14,15]. Therefore, as a preventive measure, the coagulation of lymphatic channels is necessary, even if a leakage site is not identified. If the leakage cannot be found, fibrin glue is commonly used to close potential leakages during surgery [3].

Non-surgical treatment of lymphocele developing from lymphatic injure during groin dissection is not rarely unsuccessful [6]. Owing to absence of a defined cavity, inguinal lymphocele is better resolved surgically [16]. Surgical options include lymphocele excision with either ligation of the lymphatic ducts or lymphatic-venous shunts between afferent lymphatics and the collateral branch of great saphenous vein [6].

Since its introduction in 1995, NPWT creating a subatmospheric pressure by vacuum-assisted closure therapy, has proved to be one of the most effective methods of managing all types of wounds. NPWT assists the wound healing process by increasing blood flow, removing inhibiting factors of wound healing and decreasing the bacterial count. The device consists of a polyurethane ether foam dressing or polyvinyl alcohol foam, which is adjusted to the wound cavity dimension. A perforated tube is placed within the foam aids in the evacuation of the wound discharge. The device is then covered with adhesive drape. The effluent of the wound is collected in a canister attached to the vacuum pump with adjustable negative pressure. The soiled dressing is replaced with fresh dressing, and the healing progress is assessed [17,18].

Despite NPWT has been used to treat different vascular complications, its use for lymphocele treatment has been less investigated. One study concluded that NWPT is a convenient and effective therapeutic option for lymphocele [19]. Some authors suggest that lymphorrhea after inguinal lymph nodes dissection needs NPWT [20,21]. Moreover, NPWT is accessible, easy to use, and has good patient satisfaction [19].

## 4 Conclusion

Inguinal lymphocele that is not reabsorbed or does not resolve with conservative treatment, such as drainage and compression bandaging, should be surgically treated. Lymphocele excision with ligation of lymphatic vessels, followed by NPWT appears to be a safe and effective approach.

## Funding

No funding source.

## Ethical approval

The study was approved by the local institutional review board (Hospital Beneficência Portuguesa de São Paulo, approval #3.549.142, 02/Sep/2019).

## Consent

The patient gave written informed consent for publication of his case.

## Author contribution

Caio Focassio, study concept or design, data collection, writing the paper.

Ricardo Gamboa, study concept or design, data collection, reviewing the paper.

Luis de Marco, data collection, reviewing the paper.

Daniela Fukasawa, data collection, reviewing the paper.

Talita Parente, data collection, reviewing the paper.

Vitor Dornas, study concept or design, data collection, reviewing the paper.

## Registration of research studies

Case report study, not research study.

## Guarantor

Caio C. M. Focássio.

## Provenance and peer review

Not commissioned, externally peer-reviewed.

## Declaration of Competing Interest

None.
